# Ιnnovative Health Promotion Strategies: A 6-Month Longitudinal Study on Computerized Cognitive Training for Older Adults with Minor Neurocognitive Disorders

**DOI:** 10.3390/ejihpe15030034

**Published:** 2025-03-12

**Authors:** Anna Tsiakiri, Spyridon Plakias, Pinelopi Vlotinou, Paraskevi Athanasouli, Aikaterini Terzoudi, Sotiria Kyriazidou, Aspasia Serdari, Georgia Karakitsiou, Kalliopi Megari, Nikolaos Aggelousis, Konstantinos Vadikolias, Foteini Christidi

**Affiliations:** 1 Department of Neurology, Democritus University of Thrace, 68100 Alexandroupolis, Greece; athanasoulip@gmail.com (P.A.); terzoudi@med.duth.gr (A.T.); sokyriaz@med.duth.gr (S.K.); kvadikol@med.duth.gr (K.V.); christidi.f.a@gmail.com (F.C.); 2 Department of Physical Education and Sport Science, University of Thessaly, 42100 Trikala, Greece; spyros_plakias@yahoo.gr; 3 Department of Occupational Therapy, University of West Attica, 12243 Athens, Greece; pvlotinou@uniwa.gr; 4 Department of Child and Adolescent, Democritus University of Thrace, 68100 Alexandroupolis, Greece; aserntar@med.duth.gr; 5 Department of Psychiatry, Medical School, Democritus University of Thrace, 68100 Alexandroupolis, Greece; gkarakit@med.duth.gr; 6 School of Social Sciences and Humanities, Department of Psychology, University of Western Macedonia, 53100 Florina, Greece; kmegari@psy.auth.gr; 7 Department of Physical Education and Sport Science, Democritus University of Thrace, 69100 Komotini, Greece; nagelous@phyed.duth.gr

**Keywords:** minor neurocognitive disorders, computerized cognitive training, cognitive decline, health promotion, neuroplasticity, aging

## Abstract

Minor neurocognitive disorders (NCDs) represent a transitional phase between normal cognitive aging and dementia, highlighting the importance of early interventions. This study assessed the efficacy of a structured 6-month computerized cognitive training (CCT) program in stabilizing cognitive decline among older adults with minor NCDs. One hundred participants were randomly assigned to an intervention group or a non-intervention group. The intervention group underwent weekly, personalized CCT sessions using the MeMo program, which targeted memory, attention, and adaptability. Cognitive performance was measured at baseline and after six months using the Cambridge Cognitive Examination (CAMCOG). Statistical analysis showed significant cognitive decline in the non-intervention group in orientation (*p* = 0.032), language expression (*p* = 0.008), praxis (*p* = 0.008), and memory (*p* = 0.01). In contrast, the intervention group showed no significant changes, except for a minor decline in perception (*p* = 0.003). These results suggest that CCT may help delay cognitive deterioration in minor NCDs. However, while cognitive decline was stabilized, no significant improvement was observed. Further research is recommended to investigate the long-term benefits and the transferability of cognitive gains. The findings support the use of CCT as a non-pharmacological health promotion strategy for enhancing cognitive resilience in aging populations. The novelty of this research lies in its focus on adaptive CCT as a non-pharmacological intervention, highlighting the potential role of neuroplasticity in delaying cognitive decline and offering new insights into personalized cognitive health strategies for aging populations.

## 1. Introduction

Minor neurocognitive disorders (minor NCDs), historically known as Mild Cognitive Impairment (MCI), represent a transitional stage between normal aging and dementia, characterized by noticeable but not disabling cognitive decline. Over the years, various terms have been used to describe mild cognitive deterioration, including “benign senescent forgetfulness” (Kral, 1958) and “age-associated memory impairment” (Crook & Larrabee, 1988). However, Petersen and colleagues proposed the widely accepted term MCI, which remains a benchmark in clinical and research contexts (Petersen et al., 1999). The introduction of the DSM-5 in 2013 marked another important development, broadening the concept under the category of neurocognitive disorders (NCDs), which includes both mild and major types (Simpson, 2014). Early identification of minor NCDs is essential for promoting healthy aging, as timely interventions may delay or prevent progression to severe NCDs. Epidemiological data indicate that minor NCD prevalence increases with age, affecting approximately 15.8% of individuals over 60 and 28.6% over 80 (Petersen et al., 2018). While some cases of minor NCDs revert to normal cognitive levels, others progress to dementia, with depression and genetic factors (e.g., the APOE ε4 allele) playing critical roles in determining outcomes. Thus, early detection can enhance clinical decision-making and support preventive health strategies (Liu et al., 2013; Petersen et al., 2014).

Neuroplasticity refers to the brain’s ability to reorganize and form new neural connections throughout life and plays a crucial role in maintaining cognitive function during aging (Smith, 2013). While aging is generally associated with a decline in neuroplastic potential, research highlights that the brain retains a significant capacity for adaptive changes even in later life stages (Bruel-Jungerman et al., 2007; Swaab, 1991). Factors such as cognitive stimulation, physical activity, and social engagement have been shown to enhance synaptic plasticity, promote neurogenesis, and improve overall brain resilience (Stern, 2002; Stern & Barulli, 2019). Moreover, interventions aimed at boosting neuroplasticity, including structured cognitive training and lifestyle modifications, have been linked to improved outcomes in individuals with minor NCDs (Scarmeas et al., 2003). Having an understanding of neuroplasticity during aging offers a promising path for the development of preventive strategies to slow cognitive decline and delay the onset of neurodegenerative diseases such as Alzheimer’s disease (AD) (Alexopoulos, 2005; Kolb et al., 2017; Sachs-Ericsson & Blazer, 2015). Therefore, promoting neuroplasticity through lifelong learning and a healthy lifestyle is considered essential for healthy brain aging.

Extensive research highlights the potential benefits of cognitive interventions for older adults with minor NCDs. A meta-analysis by Li et al. showed that while no pharmacological treatment provides long-term benefits, cognitive training improves both cognitive performance and self-assessment (Li et al., 2011). Finn and McDonald observed enhanced performance through computer-based training, noting that participants retained the ability to transfer skills across domains (Finn & McDonald, 2011). Similarly, Fiatarone Singh and colleagues found that combining physical exercise with cognitive training significantly improved cognitive function over 18 months (Fiatarone Singh et al., 2014). Barban and colleagues demonstrated superior outcomes from cognitive training combined with positive memory recall therapy in minor NCDs and healthy older adults across four European countries (Barban et al., 2016). Further, Hill and colleagues highlighted the advantages of computer-based interventions, including low cost, adaptability, and scalability (Hill et al., 2017). Recent studies emphasize that combined interventions, including exercise and cognitive training, are among the most effective strategies for cognitive improvement in minor NCDs (Xu et al., 2021).

Computerized cognitive training (CCT) involves the use of specially designed computerized programs, often referred to as “serious games” delivered via computers, tablets, or smart TVs. Zyda defines “serious games” as interactive digital competitions, developed with an educational goal alongside entertainment, aiming to improve skills or impart knowledge (Zyda, 2005). These games combine narrative, artistic design, and software engineering with a pedagogical dimension, emphasizing that while education is critical, entertainment should take precedence to ensure engagement. Several studies have explored the effectiveness of these programs on cognitive functions in older adults with minor NCDs (Barnes et al., 2009; Chae & Lee, 2023; Delbroek et al., 2017; Hagovská et al., 2017; Han et al., 2014; Hyer et al., 2016; Rozzini et al., 2007; Unverzagt et al., 2007). Improved cognitive functions have been reported following NPT software-based training, specifically in areas such as memory (short-term, long-term, verbal, and visual), attention (both spread and divided), perception (including perceptual recognition and identification), verbal fluency, and psychomotor learning (Cipriani et al., 2006). Similarly, Talassi et al. (Talassi et al., 2007) and Rozzini et al. (Rozzini et al., 2007) found enhanced cognitive and emotional outcomes from combined interventions, including behavioral therapy and pharmacological treatments. Barnes et al. highlighted improvements in visual memory and language skills (Barnes et al., 2009), while Herrera et al. observed positive effects on episodic recall and recognition after 12 weeks of training (Herrera et al., 2012). González-Palau and colleagues demonstrated that the “Long Lasting Memories” (LLM) program improved general cognitive function and reduced depressive symptoms over a 12-week period (González-Palau et al., 2014). Moreover, research on virtual reality-based programs has shown promising results. Thapa and colleagues and Maeng and colleagues observed cognitive improvements and enhanced visuospatial skills in participants undergoing VR-based training (Maeng et al., 2021; Thapa et al., 2020). Recent systematic reviews and meta-analyses (Bahar-Fuchs et al., 2017; Ge et al., 2018) have confirmed that CCT leads to improvements in both cognitive and emotional functioning.

Building on the extensive literature review, it becomes evident that CCT offers significant potential for promoting cognitive resilience in older adults with mild neurocognitive disorders (NCDs). However, important gaps persist regarding its long-term efficacy and the most effective implementation protocols. While existing research has predominantly focused on short-term cognitive improvements, sustained benefits and the transferability of these gains to untrained cognitive domains or everyday functional tasks remain underexplored. Therefore, the present study aims to assess the effectiveness of a structured CCT program, specifically designed to improve cognitive function and/or delay progression to severe cognitive impairment in this vulnerable population. By focusing on adaptive, computer-based interventions, this research seeks to contribute valuable insights into health promotion strategies tailored to aging individuals.

## 2. Materials and Methods

### 2.1. Subjects

All participants were regularly monitored by the interdisciplinary team of the Dementia Outpatient Clinic at the Department of Neurology, University General Hospital of Alexandroupolis, were recruited from the broader region of Thrace, and represented a diverse sample in terms of age, educational background, and reported duration of cognitive decline. All participants were initially interviewed to collect demographic data and detailed medical history, including any previous diagnoses, as well as information regarding cardiovascular, metabolic, and neurological disorders, and past or current emotional or psychiatric conditions. Following the interview, each participant underwent a thorough clinical evaluation, which included a neurological examination, cognitive assessment, brain imaging, and specialized biochemical and hematological testing. The inclusion criteria were the following: age over 50 years old; subjective cognitive decline or cognitive decline reported by an informant (e.g., spouse, adult child, or primary caregiver) who had regular contact with the participant and who could provide reliable observations of cognitive changes lasting at least six months prior to study enrollment; cognitive impairment verified through formal cognitive testing, preserved functional capacity in daily life activities; Greek as the native language; and adequate sensory and motor functions to ensure reliable participation in psychometric assessments and cognitive training sessions. Sensory and motor abilities were evaluated during the initial clinical examination, including basic vision and hearing screening (with corrective aids if necessary) and a brief neurological assessment to verify fine and gross motor skills, ensuring participants could effectively interact with the touchscreen device used in cognitive training. Cognitive impairment was verified through formal cognitive testing, including the Montreal Cognitive Assessment (MoCA) (Nasreddine et al., 2005; Tsiakiri et al., 2021) and the Cambridge Cognitive Examination (CAMCOG) (Roth et al., 1986; Tsolaki et al., 2000), in accordance with DSM-5 criteria. The diagnostic process also involved a comprehensive clinical evaluation that included medical history, neurological examination, neuroimaging, and laboratory tests to rule out secondary causes of cognitive impairment. Subjective cognitive complaints were validated through both self-reports and informant reports provided by close family members or caregivers with regular contact with the participant. To differentiate between normal cognitive aging and minor NCD, we applied neuropsychological criteria based on age- and education-adjusted normative data, considering scores 1 to 1.5 standard deviations below the mean as indicative of cognitive decline. Potential confounding factors, such as comorbid medical conditions, were systematically evaluated and considered in the diagnostic process. The exclusion criteria were the following: prior diagnosis of dementia, other neurological disease; other major psychiatric disorder (such as psychosis, major depressive disorder, bipolar disorder, etc.), major cardiovascular diseases; current pharmacological treatment with cholinesterase inhibitors, antipsychotic medications, anticholinergic drugs, benzodiazepines, or neuroleptics.

After completing the screening process, 100 individuals met the diagnostic criteria for minor NCD, as defined in the DSM-5 (Simpson, 2014), as well as the inclusion/exclusion criteria of this study, and were further allocated into the two groups of this study (i.e., an intervention group and a non-intervention group). All participants provided written informed consent prior to their inclusion in this study. Ethical approval was obtained from the Scientific Board of the University General Hospital of Alexandroupolis (ΔΣ1/Θ68/06-04-2020). The data were analyzed anonymously.

### 2.2. Materials

#### 2.2.1. Clinical Assessment

Participants’ clinical assessment and follow-up at the Dementia Outpatient Clinic were carried out by a multidisciplinary team, which consisted of experienced medical professionals, nursing staff, and experts from various other fields. All relevant information was systematically documented in an electronic medical record, including demographic details, provisional diagnoses, findings from clinical and laboratory testing, vascular risk factors, and data regarding the onset and progression of the disease.

#### 2.2.2. Neuropsychological Assessment

The primary tool used in this evaluation was the Cambridge Cognitive Examination (CAMCOG), a subscale of the Cambridge Mental Disorders of the Elderly Examination (CAMDEX), first introduced by Roth and colleagues (Roth et al., 1986; Tsolaki et al., 2000). CAMCOG is widely regarded as a reliable instrument for detecting cognitive deficits and assessing patients with minor NCDs. It provides a total score ranging from 0 to 107, based on responses to 60 individual items, and covers nine distinct cognitive domains: orientation, language comprehension, language expression, attention, praxis, calculation, abstraction, memory, and perception. The administration of the CAMCOG typically requires 30 to 40 min, but may take longer depending on the severity of the participant’s cognitive deficits. This tool has been recommended for the detection of mild NCDs and is considered effective in differentiating between normal aging and pathological cognitive decline (Aprahamian et al., 2011; Conde-Sala et al., 2012). In accordance with the study design, the assessments were conducted at two key points: initially upon the participants’ enrollment in the study (T0), and exactly six months later (T1), for both the intervention and non-intervention groups. This ensured consistency in the assessment timeline across groups, with the intervention group completing their cognitive training and the non-intervention group undergoing routine clinical follow-up during this same period.

#### 2.2.3. Computerized Cognitive Training (CTT) Program

In this study, the Memory Motivation (MeMo) program served as a primary tool for cognitive training (Memory Motivation, n.d.; P. Robert et al., 2020). The MeMo is an online application developed to support individuals with cognitive disorders, healthcare professionals engaged in the prevention of cognitive decline and stimulation, and anyone seeking to enhance their memory and attention. Designed by a team of healthcare experts from the Institut Claude Pompidou, Association IA, CoBTeK lab, and Université Côte d’Azur in Nice, France, the MeMo program was translated into Greek for the purpose of the present study (Department of Neurology, University General Hospital of Alexandroupolis, Democritus University of Thrace), following authorization from its creators. MeMo offers a variety of recreational activities and exercises specifically designed to strengthen and train cognitive functions. The program focuses on three core areas: memory, adaptability, and attention. Each exercise is tailored to target a specific cognitive skill, allowing for personalized training based on the individual’s identified deficits. The training activities in MeMo are divided into two main sections. The first section focuses on memory, offering three distinct exercises: “Recognition”, aimed at improving visual memory; “MeMo Quiz”, designed to train working memory; and “Faces”, which helps users practice memory recall. The second section emphasizes mental flexibility and attention, featuring three additional exercises: “Arrows”, which targets processing speed, inhibitory control, and cognitive flexibility; “Complex Cards”, which is geared toward enhancing working memory; and “Square Jumps”, aimed at training response prediction and inhibitory control. Each exercise includes graded difficulty levels and tracks users’ personal scores and progress over time. When a user achieves the highest score in a specific level, they are automatically promoted to the next level. The adaptive nature of the MeMo program lies in its dynamic adjustment of exercise difficulty. While the content of the exercises is standardized, the program automatically advances participants to more challenging levels when tasks are successfully completed. Conversely, if a participant struggles, the program retains them at the current level, allowing for continued practice until mastery is achieved. This adaptive mechanism ensures that each individual engages with tasks that correspond to their cognitive performance, promoting effective and personalized cognitive stimulation. After creating an account, users can monitor their performance and progress, while therapists can also track the long-term development of their patients. The MeMo application is freely accessible at the following link, http://www.memory-motivation.org/home-4/, and is available in four different languages: English, French, Italian, and Greek. It has been primarily utilized in studies conducted by its developers to evaluate its effectiveness in cognitive training for older adults with minor neurocognitive disorders. Although external studies are currently limited, the available research highlights its potential as a valuable non-pharmacological intervention.

### 2.3. Procedure

All participants were randomly assigned to one of two groups: the intervention group, consisting of participants who completed the cognitive intervention program, and The non-intervention group, which comprised individuals who continued with their standard clinical follow-up, which included routine medical monitoring, health consultations, and general advice provided by the Dementia Outpatient Clinic. However, they did not receive any structured cognitive training or specific cognitive stimulation during the study period.

For the intervention group, each individual cognitive training session consisted of activities from the MeMo program. Sessions were scheduled to last 45 min each. Sessions were held once a week over a period of six consecutive months. All interventions were tailored to individual needs, and appropriate electronic equipment was available for delivering the CCT programs. The program sessions were conducted in a dedicated space provided by the Department of Neurology, located within the urban area of the city. This environment was carefully selected to ensure minimal distractions and was equipped with adequate lighting to create an optimal setting for cognitive training. A laptop with a touchscreen was primarily used, as it was observed that using a computer mouse reduced performance in time-sensitive tasks, while tablets were deemed inadequate due to their smaller screens.

Before starting the program, participants attended two introductory sessions to become familiar with the computer equipment and software. Participation in the program was entirely voluntary, and individuals were free to withdraw at any time if they wished. The recruitment of the 50 participants was carried out in staggered phases to ensure that the program could be delivered consistently by a single researcher, who conducted all sessions to maintain uniformity and reduce the likelihood of participant dropout.

### 2.4. Statistical Analysis

Comparisons between the intervention and non-intervention groups at baseline demographic and clinical characteristics were made using an independent *t*-test (age), a Mann–Whitney U test (education, MoCA), and chi-square (sex distribution). Generalized linear mixed models (GLMMs) were applied to examine the effect of the interaction TIME*intervention on nine dependent variables of CAMCOG (orientation, language comprehension, language expression, attention, praxis, calculation, abstraction, memory, perception). A negative binomial distribution was chosen in all cases based on the Akaike Information Criterion (AIC) and Bayesian Information Criterion (BIC). The significance level was set at *p* = 0.05, adjusted for multiple comparisons with Bonferroni correction. IBM SPSS Statistics software (Version 29, IBM SPSS Inc., Chicago, IL, USA) was used for statistical analyses.

## 3. Results

The final sample consisted of 100 participants of both sexes. The demographic characteristics and MoCA scores of the participants at baseline are presented in Table 1. The groups did not differ in age (*p* = 0.172), sex distribution (*p* = 0.260), education (*p* = 0.057), or MoCA score (*p* = 0.068).

Table 2 presents descriptive statistics comparing two groups (i.e., non-intervention and intervention) at two time points, t0 (baseline) and t1 (follow-up), across various cognitive domains as measured by CAMCOG. Each cognitive domain was assessed at both time points. The results are reported separately for the groups, and the data format includes the mean value followed by the SD.

Table 3 presents the results of pairwise comparisons for the CAMCOG target variables measured at two time points (t0 vs. t1) for the two groups (the intervention and non-intervention group). Post hoc analysis revealed significant differences between the two time points (t0 vs. t1) for the non-intervention group in several CAMCOG variables, but not for the intervention group. Specifically, the non-intervention group showed significantly worse performance at t1 in orientation (*p* = 0.032), language expression (*p* = 0.008), praxis (*p* = 0.008), and memory (*p* = 0.01). Furthermore, there were significant differences for both intervention and non-intervention groups in CAMCOG-perception (intervention group: *p* = 0.003; non-intervention group, *p* = 0.03), with both groups showing worse performance at t1.

**Figure 1:**
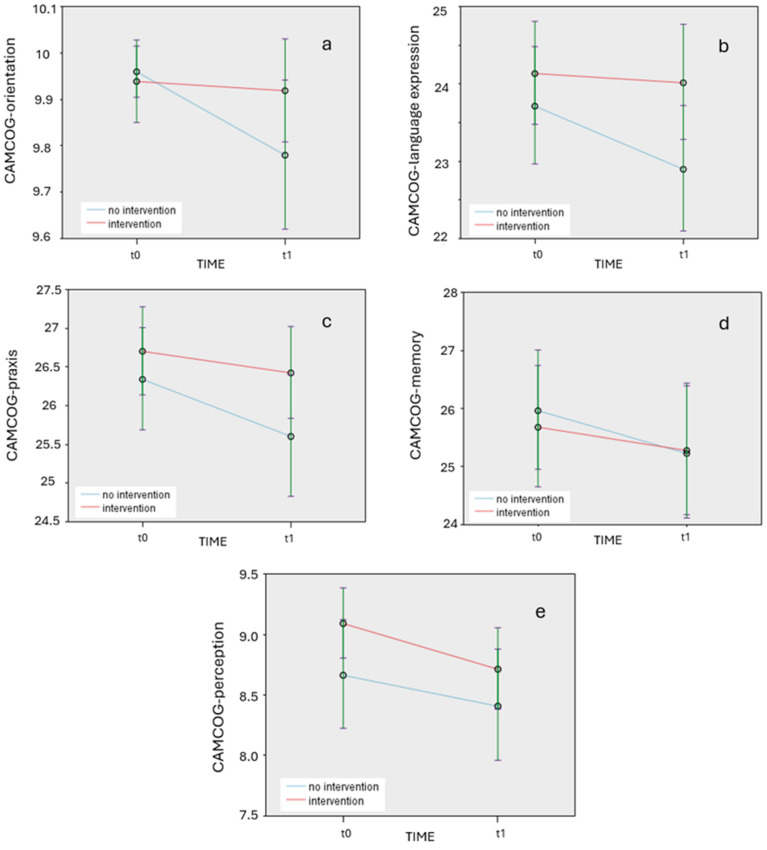
(**a**). Changes in target CAMCOG variable of orientation over time (t0 and t1) for intervention and non-intervention groups. (**b**). Changes in target CAMCOG variable of language expression over time (t0 and t1) for intervention and non-intervention groups. (**c**). Changes in target CAMCOG variable of praxis over time (t0 and t1) for intervention and non-intervention groups. (**d**). Changes in target CAMCOG variable of memory over time (t0 and t1) for intervention and non-intervention groups. (**e**). Changes in target CAMCOG variable of perception over time (t0 and t1) for intervention and non-intervention groups.

The CAMCOG variables with statistically significant differences are graphically represented in Figure 1a, Figure 1b, Figure 1c, Figure 1d, and Figure 1e, respectively.

## 4. Discussion

The present study highlights a critical distinction between the cognitive trajectories of participants receiving a 6-month intervention and those without any structured cognitive support. Specifically, our study suggests that a CCT program provided through a 6-month period may stabilize cognitive decline in individuals with minor NCD; while significant cognitive decline was observed across multiple domains in the non-intervention group, the intervention group mainly demonstrated a pattern of no changes over time.

### 4.1. CCT and Underlying Mechanisms in Cognitive Decline

One of the key findings of the present study is that overall cognitive functioning in the intervention group did not show any significant decline (except for CAMCOG-perception), a result that supports the hypothesis regarding the efficacy of the proposed personalized CCT intervention. Although no significant improvement was identified, the absence of significant decline in most cognitive domains indicates that the CCT intervention may have played a protective role against progressive cognitive deterioration. In contrast to the intervention group, the non-intervention group showed statistically significant declines in various cognitive domains. Orientation exhibited a notable reduction, reflecting diminished spatial and temporal awareness over time in the absence of external cognitive engagement. Language expression also showed a marked decline, highlighting progressive deterioration in verbal fluency and communication abilities. Similarly, praxis was negatively affected, indicating a worsening capacity to perform learned motor sequences, potentially linked to reduced coordination and planning functions. Memory performance declined as well, aligning with the expected trajectory of cognitive aging in the absence of active cognitive stimulation. Additionally, sensory perception demonstrated a significant decrease, underscoring the natural progression of cognitive impairment over time.

Our findings are in line with the existing literature suggesting that cognitive training can be a promising approach for slowing cognitive decline (Butler et al., 2018) and/or improving cognitive performance and behavioral symptoms (Ge et al., 2018; Hill et al., 2017), as well as neuropsychiatric symptoms (Liang et al., 2018). Similar trends were reported in a previous study, where the most notable changes were observed in language and memory (Barnes et al., 2009). Additionally, two other studies documented improvement tendencies in memory, although these were not supported by statistically significant results in neuropsychological tests (Rapp et al., 2002; Troyer et al., 2008). This observation aligns with previous findings, suggesting that older adults with minor NCDs can benefit from cognitive interventions across multiple cognitive and non-cognitive domains (Hughes et al., 2014).

The sustainability of cognitive improvement over time has been questioned in previous studies and cannot be answered in the present study, where only short-term effects were examined. The SMART study highlighted that cognitive training preserved memory performance, but only during the intervention period, suggesting that the benefits of cognitive interventions are contingent on continuous practice across multiple cognitive domains (Fiatarone Singh et al., 2014). Similarly, short-term improvement in cognition has been found in subjects with mild cognitive deficits following the implementation of a computer-assisted cognitive rehabilitation program (Mansbach et al., 2017). However, a systematic review of 32 studies emphasizes both short- and long-term benefits of cognitive interventions (Hong et al., 2015). Of note, CCT has been proposed as a technique with potential long-term benefits for cognitive health (Bahar-Fuchs et al., 2017; Lampit et al., 2014; Shah et al., 2017; Tsiakiri, 2022). It has been suggested that computer-based interventions are more effective for cognitive training, while structured instructional interventions are superior in fostering self-assessment skills (Li et al., 2011). CCT has also been effective for the improvement of emotional well-being. A previously published sub-study by Tsiakiri and colleagues reported similar results, showing enhancements in emotional status (Tsiakiri et al., 2024c). Furthermore, not only CCT, but also other digital activities, such as brain-training games, have been shown to enhance overall cognitive functioning (Gooding et al., 2016).

The absence of significant improvement in memory performance in the intervention group is a noteworthy finding, particularly since memory is a core domain targeted in cognitive training for individuals with NCDs. Several factors may account for this outcome. While the MeMo program includes exercises designed to stimulate memory-related processes, such as working memory and recall tasks, it is possible that the program’s structure did not provide sufficient intensity or task specificity to elicit measurable gains in memory. The adaptive design of the program focuses primarily on performance-based progression, which may favor improvements in cognitive flexibility and attention rather than engaging the deeper encoding and consolidation processes necessary for enhancing memory. Additionally, the memory tasks within the MeMo program may have been more focused on short-term and working memory rather than on episodic memory, which tends to be more vulnerable in individuals with minor NCDs. This focus could partially explain the discrepancy between our findings and those of previous studies that reported significant memory improvements following cognitive interventions specifically tailored to episodic memory, such as those incorporating spaced retrieval or associative learning techniques.

To enhance memory-related outcomes, future iterations of the program could benefit from modifications aimed at increasing task complexity with a greater emphasis on long-term memory processes. Incorporating strategy-based training approaches, such as mnemonic devices and visualization techniques, may also strengthen memory performance. Furthermore, increasing the overall training intensity and frequency, along with repeated exposure to memory-specific tasks, could contribute to more pronounced cognitive gains. Combining computerized cognitive training with real-life memory exercises or dual-task paradigms may further support the transfer of cognitive improvements to everyday memory functions.

In our study, the absence of significant cognitive decline in the intervention group, compared to the significant cognitive deterioration in several domains in the non-intervention group, support the potential of CCT in delaying progression to dementia. Although not specifically examined in the current study, the beneficial effects of cognitive training interventions are believed to result from neuroplastic changes at the synaptic level. For instance, training in sensory and motor functions has the potential to alter synaptic connections in these regions, as well as in the prefrontal cortex (Hu et al., 2021). It can also increase levels of neurotrophic factors in the brain, enhancing resistance to neural damage (Verghese et al., 2003). In addition, cognitive–neuroimaging studies support neuroplastic alterations in several brain regions following cognitive training sessions, including functional changes in the hippocampus in subjects with MCI (Rosen et al., 2011). The hippocampus is a key area for memory functions (Bartsch & Wulff, 2015), while hippocampal and medial temporal atrophy have been identified as key predictors of cognitive decline (van de Pol et al., 2007). Another study combining cognitive and neuroimaging measures in MCI subjects with and without a 7-month combined cognitive/physical training revealed significant cognitive decline in a non-intervention group and significant cognitive improvement in an intervention group, followed by a neural efficiency decline in a non-intervention group (Maffei et al., 2017). Similarly, Klados and colleagues found that cognitive training combined with physical exercise can reorganize the functional connectivity of the beta band in individuals with NCDs (Klados et al., 2016). Therefore, not only is physical exercise effective for cognitive enhancement (Pamboris et al., 2024; Vlotinou et al., 2023), but combined physical and cognitive exercises maximize the benefits for brain health by pairing physical activity and cognitive effort (Styliadis et al., 2015). The references to neuroplastic changes are grounded in findings from prior research on the effects of cognitive training in older adults, which suggest that cognitive stimulation may promote neural adaptability and support hippocampal function. However, these neurobiological mechanisms remain speculative within the context of our results. Future studies incorporating neuroimaging or biological markers would be necessary to validate such interpretations and provide direct evidence of underlying neural changes associated with CCT.

Of note, cognitive training may be more effective for patients in very early stages of cognitive impairment, with the optimal window of intervention being when individuals report subjective cognitive decline. Previous studies have shown that the early stages of Alzheimer’s disease benefit more from cognitive training (Clare et al., 2003). In contrast, reversing cognitive decline in the later stages of dementia is challenging, as deficits become severe. However, restoring residual cognitive and emotional functioning remains possible (Sakamoto et al., 2013), as the hippocampus in NCDs may retain sufficient neuroplasticity to benefit from cognitive training (Rosen et al., 2011). Therefore, considering that many subjects with minor NCDs progress to have major NCDs (Tsiakiri et al., 2024a), cognitive training interventions may have an added value by enhancing neuroplasticity and thus delaying cognitive decline in both healthy aging and NCD populations (González-Palau et al., 2014).

Although this study revealed statistically significant effects of computerized cognitive training (CCT) in preventing cognitive decline, it is important to consider whether these findings reflect clinically meaningful improvements in cognitive function. Statistical significance indicates that the observed changes are unlikely due to chance; however, this does not necessarily imply that the magnitude of change has a tangible impact on individuals’ daily functioning. The effect sizes observed in our study suggest modest improvements, particularly in domains such as memory, language, and praxis, which are crucial for maintaining functional independence in older adults. While these improvements may not represent dramatic shifts in neuropsychological test scores, even small gains in cognitive performance can be meaningful in real-world contexts, such as enhancing the ability to manage daily tasks, maintain social engagement, and reduce the risk of further cognitive deterioration. Moreover, the cumulative effect of cognitive stability over time, as observed in the intervention group, could delay progression to more severe cognitive impairment. Future research should incorporate functional outcome measures, such as activities of daily living (ADLs) and quality of life assessments, to better capture the clinical relevance of cognitive changes observed in CCT interventions.

### 4.2. Clinical Considerations and Training Effectiveness

Cognitive Training and Transfer Effects: Although the applied CCT mainly offers activities related to memory, adaptability, and attention, we found significant post-intervention differences in memory, but also in orientation, language expression, praxis, and perception. This highlights a key question in cognitive intervention research, i.e., whether improvement in a specific cognitive domain can be transferred to other untrained domains, thereby enhancing the brain’s neuroplasticity. Despite reports on limited such generalization across cognitive domains (Mansbach et al., 2017), previous studies have demonstrated that healthy older adults can benefit from cognitive training in both near-transfer tasks (similar to the trained tasks) and far-transfer tasks (dissimilar to the trained tasks) (Karbach & Kray, 2009; Willis et al., 2006). A previous meta-analysis also found significant improvement in functional abilities among individuals with minor NCDs following cognitive training, suggesting a transfer effect of cognitive training (Li et al., 2011), which can also benefit subjects’ quality of life (Hagovská et al., 2017). Of note, in other clinical groups, such as stroke, training in processing speed in particular has shown promise in improving working memory and daily functioning by maintaining two key neural networks involved in cognitive regulation: the central executive network and the default mode network (Tsiakiri et al., 2024b). The potential for transfer effects from training of one cognitive function to untrained cognitive functions holds significant clinical relevance. Two interpretations have been proposed to explain this phenomenon (Lin et al., 2016). The first posits that individuals with minor NCDs, having lower baseline cognitive abilities, possess greater room for improvement across both trained and untrained domains. The second highlights the benefits of enriched training protocols that incorporate a combination of multiple tasks.

In this study, a total of 24 sessions per participant, with a weekly frequency of one session lasting 45 min, offered over a period of 6 months, has not been clearly established in the literature as sufficient for long-term outcomes. The largest cognitive gains are thought to occur through repeated practice of a skill over an extended period, even when learned later in life. Intensive training or practice of a novel skill can alter neural structures and functions within a relatively short time frame of 1 week to 3 months (Rabipour & Raz, 2012). In another study, researchers concluded that brief sessions under 30 min may be ineffective, possibly because synaptic plasticity is more likely to occur after 30 to 60 min of stimulation (Lampit et al., 2014). On the other hand, conducting training more than three times per week might reduce the effectiveness of computer-based cognitive programs. These findings raise the possibility of an optimal “dose” of training, beyond which factors such as cognitive fatigue may diminish the benefits of the intervention. In fact, the “dose effect” of cognitive intervention is a key area of research, aimed at determining whether the effectiveness of an intervention correlates with the duration of cognitive training or the number of training sessions (Basak et al., 2008; Miller et al., 2013; Sood et al., 2019). To date, there is no strong evidence that longer sessions or a greater number of total sessions are positively associated with improved outcomes in individuals with minor NCDs. In fact, some studies with higher session counts and longer total durations reported smaller effect sizes (Li et al., 2011). Regarding the appropriate intervention duration, evidence suggests that the number of sessions may not directly influence intervention outcomes. Furthermore, a large-scale study indicated a negligible correlation between the number of training sessions and observed transfer benefits (Owen et al., 2010).

The schedule of the present study was designed to balance cognitive stimulation with feasibility, ensuring high adherence among older adults with minor neurocognitive disorders. However, the literature presents mixed findings regarding the optimal ‘dose’ of cognitive training for maximizing cognitive benefits. In fact, higher session counts and longer intervention durations do not consistently correlate with improved cognitive outcomes, with some studies reporting smaller effect sizes under more intensive protocols. Given these discrepancies, our protocol represents a moderate-intensity approach aimed at providing sufficient cognitive stimulation while maintaining long-term engagement and minimizing the risk of fatigue. Future research should focus on systematically evaluating different training frequencies, session durations, and intervention lengths to determine the most effective combination for enhancing cognitive outcomes, particularly in populations with minor NCDs.

While our study demonstrated that the intervention group maintained cognitive function over the study period, no significant cognitive improvements were observed, which contrasts with prior research reporting cognitive gains following computerized cognitive training (CCT). Several factors may account for this discrepancy. Firstly, the duration of the intervention may have influenced the outcomes. Although the six-month training period aligns with other CCT studies, it is possible that a longer intervention might be required to produce measurable cognitive gains, particularly in populations with existing cognitive impairments. Training frequency could also be a contributing factor; variations in adherence or engagement levels may have limited the cumulative cognitive stimulation necessary to drive significant improvements. Additionally, sample characteristics may have played a role. Our study focused on older adults with minor neurocognitive disorders (NCDs), a group that may have reduced neuroplasticity compared to cognitively healthy individuals, potentially limiting their capacity for cognitive gains. In contrast, studies reporting significant improvements often include younger or healthier populations with greater cognitive reserve. Finally, it is worth considering that the primary benefit of CCT in populations with minor NCD may lie in the preservation of cognitive function rather than marked improvement. Preventing decline in such vulnerable groups can be clinically meaningful, even in the absence of measurable cognitive enhancement. Future research should explore the impact of longer interventions, increased training intensity, and tailored programs to maximize cognitive outcomes.

A key limitation of this study is the lack of follow-up assessments beyond the 6-month intervention period, which limits our ability to determine the long-term sustainability of the observed cognitive benefits. While the intervention group maintained cognitive function during the study period, it remains unclear whether these effects persist over time or if cognitive decline resumes once the training is discontinued. Cognitive training interventions may produce temporary gains that diminish without continued practice, as suggested by some longitudinal studies. Conversely, sustained cognitive benefits have been reported in studies with extended follow-up periods, highlighting the potential role of booster sessions or ongoing cognitive engagement in maintaining improvements. Future research should incorporate longitudinal follow-up assessments to evaluate the durability of cognitive training effects, ideally over periods of 12 months or longer. Such studies could provide insights into the optimal frequency and duration of interventions required to achieve and maintain long-term cognitive health, particularly in populations at risk for neurocognitive decline.

The results of the present study are consistent with the existing literature on the efficacy of computerized cognitive training (CCT) in older adults with cognitive impairments. Prior studies, such as those by Smith (2013) and Lampit et al. (2014), have demonstrated that CCT interventions can lead to significant improvements in cognitive domains, including memory, attention, and executive functions. These studies emphasized the role of adaptive training protocols, where task difficulty dynamically adjusts based on the participant’s performance, thereby enhancing cognitive engagement, and promoting neuroplasticity. In line with these findings, our study employed an adaptive CCT approach through the MeMo platform, which not only adjusts the complexity of cognitive tasks in real-time, but also allows for remote, unsupervised implementation, offering greater flexibility compared to traditional, clinic-based interventions.

### 4.3. Delivery Mode in Cognitive Training

Another important aspect of the present study is the therapeutic framework in which the intervention program was delivered. It consisted exclusively of individualized, face-to-face sessions, with a personalized approach for each participant. These components have been shown to enhance the effectiveness of similar interventions (Belleville et al., 2006; Hwang et al., 2012), although some research also supports the superiority of group-based interventions (Lampit et al., 2014; Verhaeghen et al., 1992). Direct supervision by the researcher ensured adherence to the therapeutic protocol, provided encouragement for completing demanding tasks, assisted in resolving technical issues, and promoted social interaction, all of which may further contribute to the overall efficacy of such interventions.

### 4.4. Motivation in Cognitive Training

A crucial element reported in cognitive training studies is the role of motivation (Ben-Sadoun et al., 2016; Manera et al., 2015) in encouraging participation in cognitive training programs (Ben-Sadoun et al., 2018). Motivation is considered a central component of intervention theory for older adults with cognitive impairment (Savulich et al., 2017; Valentijn et al., 2005). Within the motivational framework of this program, two primary mechanisms—perceived benefit and support—were shown to influence the degree to which older adults with cognitive dysfunction are motivated to engage in exercise-based interventions. The MeMo program has been designed to enhance intrinsic motivation for continued engagement through several key features (P. Robert et al., 2020). First, the game interface is specifically tailored to older adults with cognitive dysfunction, incorporating a simplified graphical user interface, straightforward instructions with regular reminders, and clear gameplay rules, making it highly accessible to participants. Additionally, the difficulty level of the exercises is dynamically adjusted to participants’ performance, providing an error-free learning environment, and keeping them in a “challenge zone” to maintain engagement. Finally, the program is implemented on a touchscreen device to reduce barriers associated with unfamiliar technological interfaces, such as a mouse and keyboard, which older adults often find difficult to use (P. H. Robert et al., 2014).

### 4.5. Strengths and Limitations of the Study

This study presents strengths that enhance the reliability and validity of its findings. The rigorous inclusion criteria were based on DSM-5 standards for minor NCD, and the comprehensive assessments ensured accurate participant selection and minimized diagnostic bias. Additionally, the personalized CCT intervention, delivered through the MeMo application and supervised individually, promoted high engagement and adherence, resulting in minimal participant dropout. However, certain limitations must be acknowledged. The relatively small sample size and the six-month duration of the intervention may have restricted this study’s ability to detect long-term cognitive changes. Additionally, the lack of long-term follow-up makes it difficult to determine whether the observed absence of cognitive changes in the intervention group is sustainable over time. One potential limitation of this study is the absence of an active control condition for the non-intervention group. While the intervention group received structured cognitive training, the non-intervention group continued with standard clinical follow-up without any cognitive stimulation. This difference in engagement levels may introduce bias, as participants receiving active cognitive stimulation could benefit not only from the training itself, but also from increased mental activity and social interaction. Future studies should consider including an active control group engaged in non-cognitive activities (e.g., social or recreational activities) to better isolate the specific effects of computerized cognitive training. Another limitation of this study is that one of the inclusion criteria was applied by non-specialized informants, such as family members, which may somehow detract from the reliability of the sample. Furthermore, a notable limitation of this study is the geographic and cultural homogeneity of the sample, as participants were recruited exclusively from a specific region in Greece. This may limit the generalizability of the findings to broader populations with different cultural, educational, and socio-economic backgrounds. Cognitive training outcomes can be influenced by cultural factors, such as cognitive reserve, lifestyle habits, and educational systems, which may affect both baseline cognitive performance and responsiveness to interventions. Future research should aim to replicate these findings in more diverse, multicultural cohorts to enhance the external validity and applicability of computerized cognitive training (CCT) programs across different settings.

## 5. Conclusions

This longitudinal study evaluated the efficacy of a structured 6-month CCT program for older adults with minor mNCDs as part of a broader health promotion strategy. Our findings indicate that such personalized, computerized interventions can play a pivotal role in delaying cognitive decline, particularly in domains vulnerable to age-related decline, such as orientation, memory, language, and praxis. CCT aligns with health promotion principles by offering an accessible, non-pharmacological approach to enhancing mental well-being and preventing further cognitive deterioration in aging populations. Further research with larger cohorts, longer follow-up periods, and diverse populations is necessary to confirm the long-term benefits of this intervention. Additionally, integrating multimodal strategies, including physical activity and social engagement, may amplify the positive outcomes of cognitive training by addressing multiple determinants of health. These findings highlight the potential of personalized digital tools to serve as effective, scalable components of health promotion programs aimed at supporting cognitive health among older adults.

## Figures and Tables

**Table 1:** Demographic characteristics and MoCA scores of groups at baseline (t0).

	Total Sample	Non-Intervention Group	Intervention Group	*p*-Value
Age (yrs)	69.92 ± 7.45	68.90 ± 8.35	70.94 ± 6.35	0.172
Sex (M/F)	27/73	16/34	11/39	0.260
Education (yrs)	9.48 ± 4.96	8.48 ± 5.32	10.48 ± 4.39	0.057
MoCA	24.72 ± 2.07	24.40 ± 2.15	25.04 ± 1.96	0.068

Notes. yrs = years; M/F = male/female; MoCA = Montreal Cognitive Assessment.

**Table 2:** Descriptive statistics (mean ± SD) of CAMCOG scores across intervention and non-intervention groups at baseline (t0) and follow-up (t1).

	Baseline (t0) Groups		Follow-Up (t1) Groups	
	Non-Intervention	Intervention	Non-Intervention	Intervention
Orientation	9.96 ± 0.20	9.94 ± 0.31	9.78 ± 0.58	9.92 ± 0.40
Language comprehension	6.38 ± 0.75	6.44 ± 0.76	6.24 ± 0.74	6.50 ± 0.68
Language expression	23.82 ± 2.72	24.22 ± 2.35	23.00 ± 2.93	24.10 ± 2.64
Attention	6.40 ± 1.03	6.68 ± 0.65	6.46 ± 0.73	6.62 ± 0.67
Praxis	26.42 ± 2.32	26.76 ± 2.02	25.68 ± 2.80	26.48 ± 2.10
Calculation	2.02 ± 0.14	2.00 ± 0.00	2.02 ± 0.14	2.00 ± 0.00
Abstraction	5.30 ± 1.66	5.52 ± 1.52	5.42 ± 1.68	5.52 ± 1.50
Memory	26.16 ± 3.55	25.86 ± 3.60	25.42 ± 3.99	25.46 ± 3.94
Perception	8.80 ± 1.92	9.14 ± 1.05	8.54 ± 1.93	8.76 ± 1.22

Notes. SD = standard deviation; CAMCOG = Cambridge Cognitive Examination.

**Table 3:** Pairwise comparisons of CAMCOG target variables across time points (t0 vs. t1) for the two groups (non-intervention, intervention).

CAMCOG Target Variables	Groups	Contrast Estimate (SE)	Statistics (t, df, p_adj_)
Orientation	Non-intervention	0.180 (0.083)	t (196) = 2.159; **p_adj_ = 0.032**
	Intervention	0.020 (0.045)	t (196) = 0.448; p_adj_ = 0.655
Language comprehension	Non-intervention	0.139 (0.080)	t (196) = 1.749; p_adj_ = 0.082
	Intervention	−0.060 (0.071)	t (196) = −0.838; p_adj_ = 0.403
Language expression	Non-intervention	0.816 (0.304)	t (196) = 2.683; **p_adj_ = 0.008**
	Intervention	0.120 (0.228)	t (196) = 0.524; p_adj_ = 0.601
Attention	Non-intervention	−0.060 (0.130)	t (196) = −0.458; p_adj_ = 0.647
	Intervention	0.060 (0.059)	t (196) = 1.010; p_adj_ = 0.314
Praxis	Non-intervention	0.738 (0.273)	t (196) = 2.701; **p_adj_ = 0.008**
	Intervention	0.279 (0.179)	t (196) = 1.565; p_adj_ = 0.119
Calculation	Non-intervention	0.000 (0.000)	n/a
	Intervention	−0.000 (0.000)	n/a
Abstraction	Non-intervention	−0.115 (0.153)	t (196) = −0.754; p_adj_ = 0.452
	Intervention	−0.000 (0.117)	t (196) = −0.000; p_adj_ = 1.000
Memory	Non-intervention	0.734 (0.283)	t (196) = 2.592; **p_adj_ = 0.010**
	Intervention	0.397 (0.440)	t (196) = 0.902; p_adj_ = 0.368
Perception	Non-intervention	0.256 (0.117)	t (196) = 2.180; **p_adj_ = 0.030**
	Intervention	0.378 (0.125)	t (196) = 3.012; **p_adj_ = 0.003**

Notes. CAMCOG = Cambridge Cognitive Examination; SE = standard error; p_adj_ = Bonferroni adjusted *p*-value. Bold *p*-values represent significant differences between t0 and t1, after Bonferroni correction for multiple comparisons.

## Data Availability

The data are available upon request.

## References

[B1-ejihpe-15-00034] Alexopoulos G. S. (2005). Depression in the elderly. The Lancet (London, England).

[B2-ejihpe-15-00034] Aprahamian I., Diniz B. S., Izbicki R., Radanovic M., Nunes P. V., Forlenza O. V. (2011). Optimizing the CAMCOG test in the screening for mild cognitive impairment and incipient dementia: Saving time with relevant domains. International Journal of Geriatric Psychiatry.

[B3-ejihpe-15-00034] Bahar-Fuchs A., Webb S., Bartsch L., Clare L., Rebok G., Cherbuin N., Anstey K. J. (2017). Tailored and adaptive computerized cognitive training in older adults at risk for dementia: A randomized controlled trial. Journal of Alzheimer’s Disease: JAD.

[B4-ejihpe-15-00034] Barban F., Annicchiarico R., Pantelopoulos S., Federici A., Perri R., Fadda L., Carlesimo G. A., Ricci C., Giuli S., Scalici F., Turchetta C. S., Adriano F., Lombardi M. G., Zaccarelli C., Cirillo G., Passuti S., Mattarelli P., Lymperopoulou O., Sakka P., Caltagirone C. (2016). Protecting cognition from aging and Alzheimer’s disease: A computerized cognitive training combined with reminiscence therapy. International Journal of Geriatric Psychiatry.

[B5-ejihpe-15-00034] Barnes D. E., Yaffe K., Belfor N., Jagust W. J., DeCarli C., Reed B. R., Kramer J. H. (2009). Computer-based cognitive training for mild cognitive impairment: Results from a pilot randomized, controlled trial. Alzheimer Disease and Associated Disorders.

[B6-ejihpe-15-00034] Bartsch T., Wulff P. (2015). The hippocampus in aging and disease: From plasticity to vulnerability. Neuroscience.

[B7-ejihpe-15-00034] Basak C., Boot W. R., Voss M. W., Kramer A. F. (2008). Can training in a real-time strategy video game attenuate cognitive decline in older adults?. Psychology and Aging.

[B8-ejihpe-15-00034] Belleville S., Gilbert B., Fontaine F., Gagnon L., Ménard E., Gauthier S. (2006). Improvement of episodic memory in persons with mild cognitive impairment and healthy older adults: Evidence from a cognitive intervention program. Dementia and Geriatric Cognitive Disorders.

[B9-ejihpe-15-00034] Ben-Sadoun G., Manera V., Alvarez J., Sacco G., Robert P. (2018). Recommendations for the design of serious games in neurodegenerative diseases. Frontiers in Aging Neuroscience.

[B10-ejihpe-15-00034] Ben-Sadoun G., Sacco G., Manera V., Bourgeois J., König A., Foulon P., Fosty B., Bremond F., d’Arripe-Longueville F., Robert P. (2016). Physical and cognitive stimulation using an exergame in subjects with normal aging, mild and moderate cognitive impairment. Journal of Alzheimer’s Disease: JAD.

[B11-ejihpe-15-00034] Bruel-Jungerman E., Rampon C., Laroche S. (2007). Adult hippocampal neurogenesis, synaptic plasticity and memory: Facts and hypotheses. Reviews in the Neurosciences.

[B12-ejihpe-15-00034] Butler M., McCreedy E., Nelson V. A., Desai P., Ratner E., Fink H. A., Hemmy L. S., McCarten J. R., Barclay T. R., Brasure M., Davila H., Kane R. L. (2018). Does cognitive training prevent cognitive decline?: A systematic review. Annals of Internal Medicine.

[B13-ejihpe-15-00034] Chae H. J., Lee S. H. (2023). Effectiveness of online-based cognitive intervention in community-dwelling older adults with cognitive dysfunction: A systematic review and meta-analysis. International Journal of Geriatric Psychiatry.

[B14-ejihpe-15-00034] Cipriani G., Bianchetti A., Trabucchi M. (2006). Outcomes of a computer-based cognitive rehabilitation program on Alzheimer’s disease patients compared with those on patients affected by mild cognitive impairment. Archives of Gerontology and Geriatrics.

[B15-ejihpe-15-00034] Clare L., Woods R. T., Moniz Cook E. D., Orrell M., Spector A. (2003). Cognitive rehabilitation and cognitive training for early-stage Alzheimer’s disease and vascular dementia. The Cochrane Database of Systematic Reviews.

[B16-ejihpe-15-00034] Conde-Sala J. L., Garre-Olmo J., Vilalta-Franch J., Llinàs-Reglà J., Turró-Garriga O., Lozano-Gallego M., Hernández-Ferrándiz M., Pericot-Nierga I., López-Pousa S. (2012). Predictors of cognitive decline in Alzheimer’s disease and mild cognitive impairment using the CAMCOG: A five-year follow-up. International Psychogeriatrics.

[B17-ejihpe-15-00034] Crook T., Larrabee G. J. (1988). Age-associated memory impairment: Diagnostic criteria and treatment strategies. Psychopharmacology Bulletin.

[B18-ejihpe-15-00034] Delbroek T., Vermeylen W., Spildooren J. (2017). The effect of cognitive-motor dual task training with the biorescue force platform on cognition, balance and dual task performance in institutionalized older adults: A randomized controlled trial. Journal of Physical Therapy Science.

[B19-ejihpe-15-00034] Fiatarone Singh M. A., Gates N., Saigal N., Wilson G. C., Meiklejohn J., Brodaty H., Wen W., Singh N., Baune B. T., Suo C., Baker M. K., Foroughi N., Wang Y., Sachdev P. S., Valenzuela M. (2014). The Study of Mental and Resistance Training (SMART) study—Resistance training and/or cognitive training in mild cognitive impairment: A randomized, double-blind, double-sham controlled trial. Journal of the American Medical Directors Association.

[B20-ejihpe-15-00034] Finn M., McDonald S. (2011). Computerised cognitive training for older persons with mild cognitive impairment: A pilot study using a randomised controlled trial design. Brain Impairment.

[B21-ejihpe-15-00034] Ge S., Zhu Z., Wu B., McConnell E. S. (2018). Technology-based cognitive training and rehabilitation interventions for individuals with mild cognitive impairment: A systematic review. BMC Geriatrics.

[B22-ejihpe-15-00034] González-Palau F., Franco M., Bamidis P., Losada R., Parra E., Papageorgiou S. G., Vivas A. B. (2014). The effects of a computer-based cognitive and physical training program in a healthy and mildly cognitive impaired aging sample. Aging & Mental Health.

[B23-ejihpe-15-00034] Gooding A. L., Choi J., Fiszdon J. M., Wilkins K., Kirwin P. D., van Dyck C. H., Devanand D., Bell M. D., Rivera Mindt M. (2016). Comparing three methods of computerised cognitive training for older adults with subclinical cognitive decline. Neuropsychological Rehabilitation.

[B24-ejihpe-15-00034] Hagovská M., Dzvoník O., Olekszyová Z. (2017). Comparison of two cognitive training programs with effects on functional activities and quality of life. Research in Gerontological Nursing.

[B25-ejihpe-15-00034] Han J. W., Oh K., Yoo S., Kim E., Ahn K.-H., Son Y.-J., Kim T. H., Chi Y. K., Kim K. W. (2014). Development of the ubiquitous spaced retrieval-based memory advancement and rehabilitation training program. Psychiatry Investigation.

[B26-ejihpe-15-00034] Herrera C., Chambon C., Michel B. F., Paban V., Alescio-Lautier B. (2012). Positive effects of computer-based cognitive training in adults with mild cognitive impairment. Neuropsychologia.

[B27-ejihpe-15-00034] Hill N. T. M., Mowszowski L., Naismith S. L., Chadwick V. L., Valenzuela M., Lampit A. (2017). Computerized cognitive training in older adults with mild cognitive impairment or dementia: A systematic review and meta-analysis. The American Journal of Psychiatry.

[B28-ejihpe-15-00034] Hong Y. J., Jang E. H., Hwang J., Roh J. H., Lee J.-H. (2015). The efficacy of cognitive intervention programs for mild cognitive impairment: A systematic review. Current Alzheimer Research.

[B29-ejihpe-15-00034] Hu M., Wu X., Shu X., Hu H., Chen Q., Peng L., Feng H. (2021). Effects of computerised cognitive training on cognitive impairment: A meta-analysis. Journal of Neurology.

[B30-ejihpe-15-00034] Hughes T. F., Flatt J. D., Fu B., Butters M. A., Chang C.-C. H., Ganguli M. (2014). Interactive video gaming compared with health education in older adults with mild cognitive impairment: A feasibility study. International Journal of Geriatric Psychiatry.

[B31-ejihpe-15-00034] Hwang H. R., Choi S. H., Yoon D. H., Yoon B.-N., Suh Y. J., Lee D., Han I.-T., Hong C.-G. (2012). The effect of cognitive training in patients with mild cognitive impairment and early Alzheimer’s disease: A preliminary study. Journal of Clinical Neurology (Seoul, Korea).

[B32-ejihpe-15-00034] Hyer L., Scott C., Atkinson M. M., Mullen C. M., Lee A., Johnson A., Mckenzie L. C. (2016). Cognitive training program to improve working memory in older adults with MCI. Clinical Gerontologist.

[B33-ejihpe-15-00034] Karbach J., Kray J. (2009). How useful is executive control training? Age differences in near and far transfer of task-switching training. Developmental Science.

[B34-ejihpe-15-00034] Klados M. A., Styliadis C., Frantzidis C. A., Paraskevopoulos E., Bamidis P. D. (2016). Beta-band functional connectivity is reorganized in mild cognitive impairment after combined computerized physical and cognitive training. Frontiers in Neuroscience.

[B35-ejihpe-15-00034] Kolb B., Harker A., Gibb R. (2017). Principles of plasticity in the developing brain. Developmental Medicine and Child Neurology.

[B36-ejihpe-15-00034] Kral V. A. (1958). Senescent memory decline and senile amnestic syndrome. The American Journal of Psychiatry.

[B37-ejihpe-15-00034] Lampit A., Hallock H., Valenzuela M. (2014). Computerized cognitive training in cognitively healthy older adults: A systematic review and meta-analysis of effect modifiers. PLoS Medicine.

[B38-ejihpe-15-00034] Li H., Li J., Li N., Li B., Wang P., Zhou T. (2011). Cognitive intervention for persons with mild cognitive impairment: A meta-analysis. Ageing Research Reviews.

[B39-ejihpe-15-00034] Liang J., Xu Y., Lin L., Jia R., Zhang H., Hang L. (2018). Comparison of multiple interventions for older adults with Alzheimer disease or mild cognitive impairment. Medicine.

[B40-ejihpe-15-00034] Lin F., Heffner K. L., Ren P., Tivarus M. E., Brasch J., Chen D.-G., Mapstone M., Porsteinsson A. P., Tadin D. (2016). Cognitive and neural effects of vision-based speed-of-processing training in older adults with amnestic mild cognitive impairment: A pilot study. Journal of the American Geriatrics Society.

[B41-ejihpe-15-00034] Liu C.-C., Kanekiyo T., Xu H., Bu G. (2013). Apolipoprotein E and Alzheimer disease: Risk, mechanisms and therapy. Nature Reviews Neurology.

[B42-ejihpe-15-00034] Maeng S., Hong J. P., Kim W.-H., Kim H., Cho S.-E., Kang J. M., Na K.-S., Oh S.-H., Park J. W., Bae J. N., Cho S.-J. (2021). Effects of virtual reality-based cognitive training in the elderly with and without mild cognitive impairment. Psychiatry Investigation.

[B43-ejihpe-15-00034] Maffei L., Picano E., Andreassi M. G., Angelucci A., Baldacci F., Baroncelli L., Begenisic T., Bellinvia P. F., Berardi N., Biagi L., Bonaccorsi J., Bonanni E., Bonuccelli U., Borghini A., Braschi C., Broccardi M., Bruno R. M., Caleo M., Carlesi C., Train the Brain Consortium (2017). Randomized trial on the effects of a combined physical/cognitive training in aged MCI subjects: The Train the Brain study. Scientific Reports.

[B44-ejihpe-15-00034] Manera V., Petit P.-D., Derreumaux A., Orvieto I., Romagnoli M., Lyttle G., David R., Robert P. H. (2015). “Kitchen and cooking”, a serious game for mild cognitive impairment and Alzheimer’s disease: A pilot study. Frontiers in Aging Neuroscience.

[B45-ejihpe-15-00034] Mansbach W. E., Mace R. A., Clark K. M. (2017). The efficacy of a computer-assisted cognitive rehabilitation program for patients with mild cognitive deficits: A pilot study. Experimental Aging Research.

[B46-ejihpe-15-00034] Memory Motivation (n.d.). Retrieved 10 February 2025. https://www.memory-motivation.org/home-4/.

[B47-ejihpe-15-00034] Miller K. J., Dye R. V., Kim J., Jennings J. L., O’Toole E., Wong J., Siddarth P. (2013). Effect of a computerized brain exercise program on cognitive performance in older adults. The American Journal of Geriatric Psychiatry: Official Journal of the American Association for Geriatric Psychiatry.

[B48-ejihpe-15-00034] Nasreddine Z. S., Phillips N. A., Bédirian V., Charbonneau S., Whitehead V., Collin I., Cummings J. L., Chertkow H. (2005). The montreal cognitive assessment, MoCA: A brief screening tool for mild cognitive impairment. Journal of the American Geriatrics Society.

[B49-ejihpe-15-00034] Owen A. M., Hampshire A., Grahn J. A., Stenton R., Dajani S., Burns A. S., Howard R. J., Ballard C. G. (2010). Putting brain training to the test. Nature.

[B50-ejihpe-15-00034] Pamboris G. M., Plakias S., Tsiakiri A., Karakitsiou G., Bebeletsi P., Vadikolias K., Aggelousis N., Tsiptsios D., Christidi F. (2024). Physical therapy in neurorehabilitation with an emphasis on sports: A bibliometric analysis and narrative review. Sports.

[B51-ejihpe-15-00034] Petersen R. C., Caracciolo B., Brayne C., Gauthier S., Jelic V., Fratiglioni L. (2014). Mild cognitive impairment: A concept in evolution. Journal of Internal Medicine.

[B52-ejihpe-15-00034] Petersen R. C., Lopez O., Armstrong M. J., Getchius T. S. D., Ganguli M., Gloss D., Gronseth G. S., Marson D., Pringsheim T., Day G. S., Sager M., Stevens J., Rae-Grant A. (2018). Practice guideline update summary: Mild cognitive impairment: Report of the guideline development, dissemination, and implementation subcommittee of the american academy of neurology. Neurology.

[B53-ejihpe-15-00034] Petersen R. C., Smith G. E., Waring S. C., Ivnik R. J., Tangalos E. G., Kokmen E. (1999). Mild cognitive impairment: Clinical characterization and outcome. Archives of Neurology.

[B54-ejihpe-15-00034] Rabipour S., Raz A. (2012). Training the brain: Fact and fad in cognitive and behavioral remediation. Brain and Cognition.

[B55-ejihpe-15-00034] Rapp S., Brenes G., Marsh A. P. (2002). Memory enhancement training for older adults with mild cognitive impairment: A preliminary study. Aging & Mental Health.

[B57-ejihpe-15-00034] Robert P., Manera V., Derreumaux A., Ferrandez Y Montesino M., Leone E., Fabre R., Bourgeois J. (2020). Efficacy of a web app for cognitive training (MeMo) regarding cognitive and behavioral performance in people with neurocognitive disorders: Randomized controlled trial. Journal of Medical Internet Research.

[B56-ejihpe-15-00034] Robert P. H., König A., Amieva H., Andrieu S., Bremond F., Bullock R., Ceccaldi M., Dubois B., Gauthier S., Kenigsberg P.-A., Nave S., Orgogozo J. M., Piano J., Benoit M., Touchon J., Vellas B., Yesavage J., Manera V. (2014). Recommendations for the use of serious games in people with Alzheimer’s disease, related disorders and frailty. Frontiers in Aging Neuroscience.

[B58-ejihpe-15-00034] Rosen A. C., Sugiura L., Kramer J. H., Whitfield-Gabrieli S., Gabrieli J. D. (2011). Cognitive training changes hippocampal function in mild cognitive impairment: A pilot study. Journal of Alzheimer’s Disease: JAD.

[B59-ejihpe-15-00034] Roth M., Tym E., Mountjoy C. Q., Huppert F. A., Hendrie H., Verma S., Goddard R. (1986). CAMDEX. A standardised instrument for the diagnosis of mental disorder in the elderly with special reference to the early detection of dementia. The British Journal of Psychiatry: The Journal of Mental Science.

[B60-ejihpe-15-00034] Rozzini L., Costardi D., Chilovi B. V., Franzoni S., Trabucchi M., Padovani A. (2007). Efficacy of cognitive rehabilitation in patients with mild cognitive impairment treated with cholinesterase inhibitors. International Journal of Geriatric Psychiatry.

[B61-ejihpe-15-00034] Sachs-Ericsson N., Blazer D. G. (2015). The new DSM-5 diagnosis of mild neurocognitive disorder and its relation to research in mild cognitive impairment. Aging & Mental Health.

[B62-ejihpe-15-00034] Sakamoto M., Ando H., Tsutou A. (2013). Comparing the effects of different individualized music interventions for elderly individuals with severe dementia. International Psychogeriatrics.

[B63-ejihpe-15-00034] Savulich G., Piercy T., Fox C., Suckling J., Rowe J. B., O’Brien J. T., Sahakian B. J. (2017). Cognitive training using a novel memory game on an ipad in patients with amnestic mild cognitive impairment (aMCI). International Journal of Neuropsychopharmacology.

[B64-ejihpe-15-00034] Scarmeas N., Zarahn E., Anderson K. E., Hilton J., Flynn J., Van Heertum R. L., Sackeim H. A., Stern Y. (2003). Cognitive reserve modulates functional brain responses during memory tasks: A PET study in healthy young and elderly subjects. NeuroImage.

[B65-ejihpe-15-00034] Shah T. M., Weinborn M., Verdile G., Sohrabi H. R., Martins R. N. (2017). Enhancing cognitive functioning in healthly older adults: A systematic review of the clinical significance of commercially available computerized cognitive training in preventing cognitive decline. Neuropsychology Review.

[B66-ejihpe-15-00034] Simpson J. R. (2014). DSM-5 and neurocognitive disorders. The Journal of the American Academy of Psychiatry and the Law.

[B67-ejihpe-15-00034] Smith G. S. (2013). Aging and neuroplasticity. Dialogues in Clinical Neuroscience.

[B68-ejihpe-15-00034] Sood P., Kletzel S. L., Krishnan S., Devos H., Negm A., Hoffecker L., Machtinger J., Hu X., Heyn P. C. (2019). Nonimmersive brain gaming for older adults with cognitive impairment: A scoping review. The Gerontologist.

[B69-ejihpe-15-00034] Stern Y. (2002). What is cognitive reserve? Theory and research application of the reserve concept. Journal of the International Neuropsychological Society: JINS.

[B70-ejihpe-15-00034] Stern Y., Barulli D. (2019). Cognitive reserve. Handbook of Clinical Neurology.

[B71-ejihpe-15-00034] Styliadis C., Kartsidis P., Paraskevopoulos E., Ioannides A. A., Bamidis P. D. (2015). Neuroplastic effects of combined computerized physical and cognitive training in elderly individuals at risk for dementia: An eLORETA controlled study on resting states. Neural Plasticity.

[B72-ejihpe-15-00034] Swaab D. F. (1991). Brain aging and Alzheimer’s disease, “Wear and tear” versus “Use it or lose it”. Neurobiology of Aging.

[B73-ejihpe-15-00034] Talassi E., Guerreschi M., Feriani M., Fedi V., Bianchetti A., Trabucchi M. (2007). Effectiveness of a cognitive rehabilitation program in mild dementia (MD) and mild cognitive impairment (MCI): A case control study. Archives of Gerontology and Geriatrics.

[B74-ejihpe-15-00034] Thapa N., Park H. J., Yang J.-G., Son H., Jang M., Lee J., Kang S. W., Park K. W., Park H. (2020). The effect of a virtual reality-based intervention program on cognition in older adults with mild cognitive impairment: A randomized control trial. Journal of Clinical Medicine.

[B75-ejihpe-15-00034] Troyer A. K., Murphy K. J., Anderson N. D., Moscovitch M., Craik F. I. M. (2008). Changing everyday memory behaviour in amnestic mild cognitive impairment: A randomised controlled trial. Neuropsychological Rehabilitation.

[B76-ejihpe-15-00034] Tsiakiri A. (2022). Remote learning for adults with mild cognitive impairment in the new landscape of COVID-19 restrictions. Advances in Social Sciences Research Journal.

[B77-ejihpe-15-00034] Tsiakiri A., Bakirtzis C., Plakias S., Vlotinou P., Vadikolias K., Terzoudi A., Christidi F. (2024a). Predictive models for the transition from mild neurocognitive disorder to major neurocognitive disorder: Insights from clinical, demographic, and neuropsychological data. Biomedicines.

[B78-ejihpe-15-00034] Tsiakiri A., Christidi F., Tsiptsios D., Vlotinou P., Kitmeridou S., Bebeletsi P., Kokkotis C., Serdari A., Tsamakis K., Aggelousis N., Vadikolias K. (2024b). Processing speed and attentional shift/mental flexibility in patients with stroke: A comprehensive review on the trail making test in stroke studies. Neurology International.

[B79-ejihpe-15-00034] Tsiakiri A., Ioannidis P., Vlotinou P., Kokkotis C., Megagianni S., Toumaian M., Terzoudi K., Koutzmpi V., Despoti A., Megari K., Liozidou A., Kyriazidoy S., Vadikolias K., Tsapanou A. (2024c). The role of a computerized cognitive intervention program on the neuropsychiatric symptoms in mild cognitive impairment. Aging Medicine and Healthcare.

[B80-ejihpe-15-00034] Tsiakiri A., Vadikolias K., Tripsianis G., Vlotinou P., Serdari A., Terzoudi A., Heliopoulos I. (2021). Influence of social and demographic factors on the montreal cognitive assessment (MoCA) test in rural population of North-Eastern Greece. Geriatrics.

[B81-ejihpe-15-00034] Tsolaki M., Fountoulakis K. N., Chantzi H., Kazis A. (2000). The Cambridge Cognitive Examination (CAMCOG): A validation study in outpatients suffering from dementia and non-demented elderly subjects (including Age Associated Cognitive Decline patients) in Greece. American Journal of Alzheimer’s Disease & Other Dementias^®^.

[B82-ejihpe-15-00034] Unverzagt F. W., Kasten L., Johnson K. E., Rebok G. W., Marsiske M., Koepke K. M., Elias J. W., Morris J. N., Willis S. L., Ball K., Rexroth D. F., Smith D. M., Wolinsky F. D., Tennstedt S. L. (2007). Effect of memory impairment on training outcomes in ACTIVE. Journal of the International Neuropsychological Society.

[B83-ejihpe-15-00034] Valentijn S. A. M., van Hooren S. A. H., Bosma H., Touw D. M., Jolles J., van Boxtel M. P. J., Ponds R. W. H. M. (2005). The effect of two types of memory training on subjective and objective memory performance in healthy individuals aged 55 years and older: A randomized controlled trial. Patient Education and Counseling.

[B84-ejihpe-15-00034] van de Pol L. A., Korf E. S. C., van der Flier W. M., Brashear H. R., Fox N. C., Barkhof F., Scheltens P. (2007). Magnetic resonance imaging predictors of cognition in mild cognitive impairment. Archives of Neurology.

[B85-ejihpe-15-00034] Verghese J., Lipton R. B., Katz M. J., Hall C. B., Derby C. A., Kuslansky G., Ambrose A. F., Sliwinski M., Buschke H. (2003). Leisure activities and the risk of dementia in the elderly. The New England Journal of Medicine.

[B86-ejihpe-15-00034] Verhaeghen P., Marcoen A., Goossens L. (1992). Improving memory performance in the aged through mnemonic training: A meta-analytic study. Psychology and Aging.

[B87-ejihpe-15-00034] Vlotinou P., Tsiakiri A., Frantzidis C. A., Katsouri I.-G., Aggelousis N. (2023). The effect of an interventional movement program on the mechanical gait characteristics of a patient with dementia. Engineering Proceedings.

[B88-ejihpe-15-00034] Willis S. L., Tennstedt S. L., Marsiske M., Ball K., Elias J., Koepke K. M., Morris J. N., Rebok G. W., Unverzagt F. W., Stoddard A. M., Wright E., ACTIVE Study Group (2006). Long-term effects of cognitive training on everyday functional outcomes in older adults. JAMA.

[B89-ejihpe-15-00034] Xu Z., Sun W., Zhang D., Chung V. C.-H., Sit R. W.-S., Wong S. Y.-S. (2021). Comparative effectiveness of interventions for global cognition in patients with mild cognitive impairment: A systematic review and network meta-analysis of randomized controlled trials. Frontiers in Aging Neuroscience.

[B90-ejihpe-15-00034] Zyda M. (2005). From visual simulation to virtual reality to games. Computer.

